# Chronic Manganese Administration with Longer Intervals Between Injections Produced Neurotoxicity and Hepatotoxicity in Rats

**DOI:** 10.1007/s11064-020-03059-2

**Published:** 2020-06-02

**Authors:** Xi-Min Fan, Ying Luo, Yu-Ming Cao, Ting-Wang Xiong, Sheng Song, Jie Liu, Qi-Yuan Fan

**Affiliations:** 1 School of Public Health, Zunyi Medical University, Zunyi, China; 2 Zunyi Medical and Pharmaceutical College, Zunyi, China; 3 University of North Carolina at Chapel Hill, Chapel Hill, NC USA; 4 Key Lab for Basic Pharmacology of Ministry of Education, Zunyi Medical University, Zunyi, China; 5 The First People’s Hospital of Bijie City, Bijie, China; 6 The Third Affiliated Hospital of Zunyi Medical University, Zunyi, China

**Keywords:** Chronic manganism, Substantia nigra pars compacta (SNpc), Microglia, Dopaminergic neuron loss, Liver inflammation, Hepatic transporters

## Abstract

**Abstract:**

Subacute exposure to manganese (Mn) produced Parkinson’s disease-like syndrome called Manganism. Chronic onset and progression are characteristics of Manganism, therefore, this study aimed to examine Mn toxicity following chronic exposures. Male Sprague-Dawley rats were injected Mn^2+^ 1 and 5 mg/kg, every 10 days for 150 days (15 injections). Animal body weight and behavioral activities were recorded. At the end of experiments, the brain and liver were collected for morphological and molecular analysis. Chronic Mn exposure did not affect animal body weight gain, but the high dose of Mn treatment caused 20% mortality after 140 days of administration. Motor activity deficits were observed in a dose-dependent manner at 148 days of Mn administration. Immunofluorescence double staining of substantia nigra pars compacta (SNpc) revealed the activation of microglia and loss of dopaminergic neurons. The chronic neuroinflammation mediators TNFα, inflammasome Nlrp3, Fc fragment of IgG receptor IIb, and formyl peptide receptor-1 were increased, implicating chronic Mn-induced neuroinflammation. Chronic Mn exposure also produced liver injury, as evidenced by hepatocyte degeneration with pink, condensed nuclei, indicative of apoptotic lesions. The inflammatory cytokines TNFα, IL-1β, and IL-6 were increased, alone with stress-related genes heme oxygenase-1, NAD(P)H:quinone oxidoreductase-1 and metallothionein. Hepatic transporters, such as multidrug resistant proteins (Abcc1, Abcc2, and Abcc3) and solute carrier family proteins (Slc30a1, Slc39a8 and Slc39a14) were increased in attempt to eliminate Mn from the liver. In summary, chronic Mn exposure produced neuroinflammation and dopaminergic neuron loss in the brain, but also produced inflammation to the liver, with upregulation of hepatic transporters.

**Graphic Abstract:**

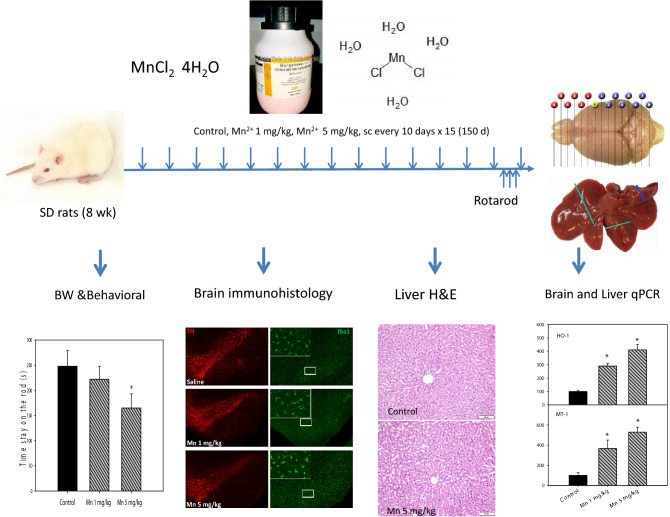

**Electronic supplementary material:**

The online version of this article (10.1007/s11064-020-03059-2) contains supplementary material, which is available to authorized users.

## Introduction

Manganese (Mn) is widely used in modern industries. Occupational exposures to Mn occur primarily through the steel industry, smelters and welding [4, 10, 15, 24]. Overexposure to Mn is known to cause clinical syndromes, including the neurologic disorder like Parkinsonism, dystonia, neuropsychiatric symptoms, motor activity impairment, subclinical liver dysfunction and increased serum bilirubin levels [14, 27, 34, 40]. Mn neurotoxicity is known to specifically damage the basal ganglia in humans [1, 34, 35]. Chronic exposure to Mn results in Manganism, a disorder displays nonmotor dysfunction and motor impairment that resembling, but not identical to Parkinson’s disease (PD, [4, 35].

Intrastriatal injection of MnCl_2_ into rats produces acute Manganism, with reduction of dopaminergic neurons, activation of microglia, and elevation of inflammatory cytokines [44]. Subacute intraperitoneal injections of MnCl_2_ or MnO_2_ are often used as models to study neurotoxic effects of Mn. For example, high dose of MnCl_2_ injections (100 mg/kg, for 3–7 days) produced oxidative damage, mitochondria dysfunction, and neuroinflammation [28],moderate dose of MnCl_2_ injections (6–15 mg/kg, 5/week for 4 weeks) altered brain dopamine levels and disrupted dopamine metabolism [30], and low dose of MnCl_2_ (2.5 mg/kg) or MnO_2_ (1.22 mg/kg) injections for 8–12 weeks altered brain neurotransmitters including decreased dopamine and its metabolites [29]. MnCl_2_ (15–50 mg/kg) injections every 2 days for 12 weeks reduced tyrosine hydrolase (TH) expression in a dose-dependent manner [43]. Given the facts that Mn has a relatively shorter half-life in blood, yet fairly long half-lives in tissues [31] and chronic progressive feature of Manganism and PD, a longer recovery time between injections, and longer observation periods are desired to define chronic toxicity effects of Mn exposure.

Manganism and hepatic encephalopathy are the most common pathologies from Mn exposures [35]. Liver dysfunction and injury are frequently observed in Mn exposed workers [4, 14, 35, 40], and Manganism patients are often associated with liver diseases [23]. Chronic liver diseases can result in an excessive accumulation of Mn in brain with ensuing signs and symptoms clinically called Mn hepatic encephalopathy [27]. With weakened liver function, there is also an increased risk of neurodegeneration with continued Mn exposure [40]. Experimentally, subacute intravenous MnCl_2_ administration to dogs caused severe hepatotoxicity with periportal hemorrhage, hepatocellular necrosis, and biliary epithelial hyperplasia [18],oral MnCl_2_ (20 mg/ml via the drinking water) to rats for 30 days increased serum enzyme activities and oxidative damage with reduced antioxidants, resulting in DNA fragmentation, apoptosis and necrosis [8]. Injections of MnCl_2_ (6 mg/kg for 30–90 days) to rats produced liver injury with mitochondria dysfunction [16]. Mn is thought to be metabolized and removed by the liver [23, 35). Hepatobiliary excretion of Mn represents a primary route of Mn clearance from the body, accounting for 80% of Mn elimination [19, 31]. Thus, the normal liver function is essential to maintain homeostasis of Mn in the body, including the brain.

Occupational Mn exposure occurs in Zunyi, China. In collaboration with Purdue University, we have conducted studies of Mn smelter workers on early behavioral changes and biomarkers, and identified the blood Mn/Fe ratio as a novel biomarker of Mn exposure [9, 10], we have discovered the association of divalent metal transporter-1, transferrin and hepcidin in blood and Mn exposures [12], and demonstrated reduced *PARK2* expression in Mn smelter workers [13]. A transportable in vivo neutron activation analysis system was designed and utilized to assess bone Mn as accumulative Mn exposure, and found bone Mn and fingurenail Mn are associated with cognitive dysfunction in Mn-exposed workers [36, 37]. In conjunction with these human studies, we have also performed experimental studies on Mn toxicity following oral [38] and peripheral exposures [25]. Subacute Mn injections produced motor deficits and brain damage, but also disrupted circadian clock in the hippocampus and liver [25]. The present study further used the low-dose (Mn^2+^ 1 and 5 mg/kg), long recovery intervals between injections (every 10 days) for a longer period of exposure (150 days) to examine chronic toxicity of Mn exposure to the brain and liver to provide a whole picture for better understanding chronic Mn exposure and health effects.

## Material and Method

### Reagent

Manganese(II) chloride tetrahydrate (MnCl_2_·4H_2_O) was purchased from Sigma Chemical Co. (St. Louis, USA) and was dissolved in normal saline. All other chemicals were of reagent grade. Antibodies against tyrosine hydroxylase (TH, rabbit: AB152) was from EMD Millipore (Temecula, CA), and against Iba-1 was from Wako Chemicals (Richmond, VA).

### Animals and Treatment

Adult male Sprague-Dawley rats, weighting 220 ± 15 g, were purchased from the Experimental Animal Center of Third Military Medical College (Chongqing, China) and maintained in the special pathogen free (SPF)-grade animal facilities at the Key Lab of Pharmacology of Minister of Education at Zunyi Medical University. Rats were fed standard rodent chow and drinking water with 21 ± 2 °C and the light on from 8:00 to 20:00. Animal studies were conducted in accordance with the Chinese Guidelines of Animal Care and Welfare. The protocol was approved by the Zunyi Medical College Animal Care and Use Committee (2015-11).

Thirty rats were randomly divided into three groups (n = 10). The low dose group was given Mn^2+^ 1 mg/kg, converted to MnCl_2_·4H_2_O of 3.6 mg/kg; and the high dose group was given Mn^2+^ 5 mg/kg, converted to MnCl_2_·4H_2_O as 18 mg/kg, and control group was injected with the same volume of saline. Rats were intraperitoneal injected every 10 days for 150 days (15 injections in total). The dose of Mn selection was based on our recent publication [25]. The experimental design is illustrated below.
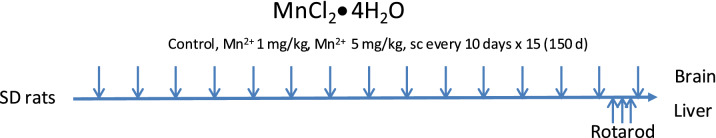


### Animal Body Weight and Rotarod Test

Animal body weights were recorded every 10 days after Mn administration. The general health, animal behavioral activity and mortality were monitored. Rotarod test was conducted to evaluate the damage of Mn on motor function. Rats were placed on the rotating rod accelerating from 10 to 40 rpm (increasing 10 rotations/min every 30 s) until the fall from the rod [25]. Each rat was subject to training for three days. The data of 148 days after Mn^2+^ administration was used for calculation.

### Tissue Collection

Twenty-four hours after the last dose (150 days from starting Mn administration), rats were euthanized with 6% choral hydrate. Under deep anesthesia, rats (3 per group) were transcardially perfused with PBS, followed by perfusion with 4% paraformaldehyde (PFA). Brains were further post-fixed in 4% PFA for 24 h, then transferred to ice-cold 10% sucrose solution and incubated at 4 °C overnight. The 10% sucrose was then replaced with ice-cold 30% sucrose, and brains were allowed to equilibrate completely at 4 °C, as evidenced by their sinking to the bottom of the container. Brains were then sectioned immediately on a freezing sliding microtome and processed for immune-staining as described previously [39]. The brains and livers of the remaining rats were removed, and SNpc-enriched midbrain was isolated and snap-frozen in liquid nitrogen and stored at − 80 °C until analysis.

### Immunohistochemistry

Free-floating 35 μm coronal sections containing the SNpc were cut on a horizontal sliding microtome. A total of 8 sections were sampled at 105 µm intervals for each brain region. The free-floating sections were immune-blocked with 4% goat serum in 0.25% Triton/PBS for 2 h and then incubated with Iba-1 antibody (1:2000, Wako Chemicals, Richmond, VA) overnight at 4 °C. On the second day, the sections were washed by 1% BSA in 0.25% Triton/PBS before the incubation with anti-tyrosine hydroxylase (TH) antibody (1:2000, EMD Millipore, Temecula, CA) overnight at 4 °C. The double-label immunofluorescence pictures were taken under the confocal microscope by using Alexa-488 (green) and Alexa-594 (red) conjugated secondary antibodies (1:1000) to visualize the TH immune reactive (THir) or Iba-1 positive cells. Stereological counts of TH+ SNpc neurons were estimated using an optical fractionator method on an Olympus BX50 stereological microscope within user-defined boundaries. Samples were countered in a double-blind manner. Data were expressed as percentage to saline-injected controls [26, 39].

### Histopathology

Liver samples were fixed in 10% buffered formaldehyde for 48 h, and processed with standard histological procedures. Livers were embedded in paraffin cassettes, and cut into 4 µm slices by using a RM2235 microtome (Leica, Germany), and stained with hematoxylin and eosin (H&E) for blinded examination under a light microscope (Olympus, Japan).

### RNA Isolation and qPCR Analysis

Real-time qPCR analysis was performed as described [26]. Briefly, total RNA was extracted with TRIzol and reverse transcribed to cDNA with TaKaRa RT kits (Dalian, China). Real-time PCR was performed with the SYBR Green PCR Kit (Bio-Rad Laboratories, Hercules, CA, USA) on Bio-Rad CFX-96 real-time PCR system. The primers were designed with Primer3 software and listed in Supplementary Table 1. The Ct values were used to calculate the relative expression by the 2^−ΔΔCt^ method and normalized with β-actin, setting control as 100%.

### Statistical Analysis

Data were expressed as mean ± SEM. Comparisons were made using one-way ANOVA analysis followed by the Statistical Package for Social Sciences (SPSS) software (version 17.0). The significant criteria were set at p < 0.05.

## Results

### General Health and Animal Body Weights

Adult male SD rats were injected saline, Mn^2+^ 1 mg and 5 mg/kg, every 10 days for 150 days. Animal body weights were recorded every 10 days after Mn administration. No apparent differences in body weights were observed during the treatment period. At the end of 150 days of administration, the body weights were 369 ± 15, 391 ± 13, and 387 ± 9 for Control, Mn^2+^ 1 mg/kg, Mn^2+^ 5 mg/kg, respectively. Two rats of Mn^2+^ 5 mg/kg group died after 140 days of administration (mortality rate 20%), and the general activity of the high dose group decreased at the end of experiment. The general health of Mn^2+^ 1 mg/kg group was not different from Controls. In the previous repeated Mn intoxication study (30 injections in 30 days), Mn accumulation in the brain was 520, 594, and 811 ng/g in Control, Mn^2+^ 1 mg and Mn^2+^ 5 mg/kg groups, respectively; Mn accumulation in the liver was 1179, 1216, and 1726 ng/g in Control, Mn^2+^ 1 mg and Mn^2+^ 5 mg/kg groups, respectively [25].

### Chronic Mn Treatment Impaired Motor Activity

**Fig. 1:**
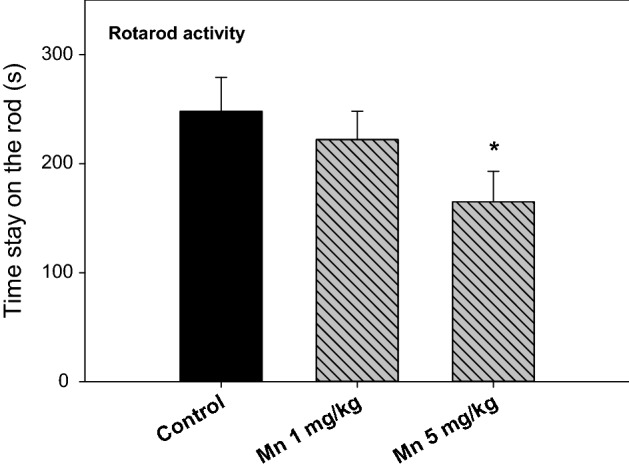
Behavioral (Rotarod, time stay on the rod) tests. Rats were given Mn^2+^ 1 mg/kg, 5 mg/kg every 10 days. Rotarod tests were performed at 148 days after Mn administration (14 injections). Data are mean + SEM of 8–10 rats. *Significantly different from Controls, p < 0.05

The behavioral activities were examined at 148 days after Mn administration. Rotarod test is one of classic methods to measure the muscular coordination in PD animals. As shown in Fig. 1, Mn administration decreased Rotarod activity, especially in the high dose group. The time stay on the rod was 248 ± 31, 222 ± 26, and 165 ± 28 for Control (n = 10), Mn^2+^ 1 mg/kg (n = 10), and Mn^2+^ 5 mg/kg (n = 8), respectively.

### Chronic Mn Treatment Produced Neuroinflammation and Dopaminergic Neuron Loss

**Fig. 2:**
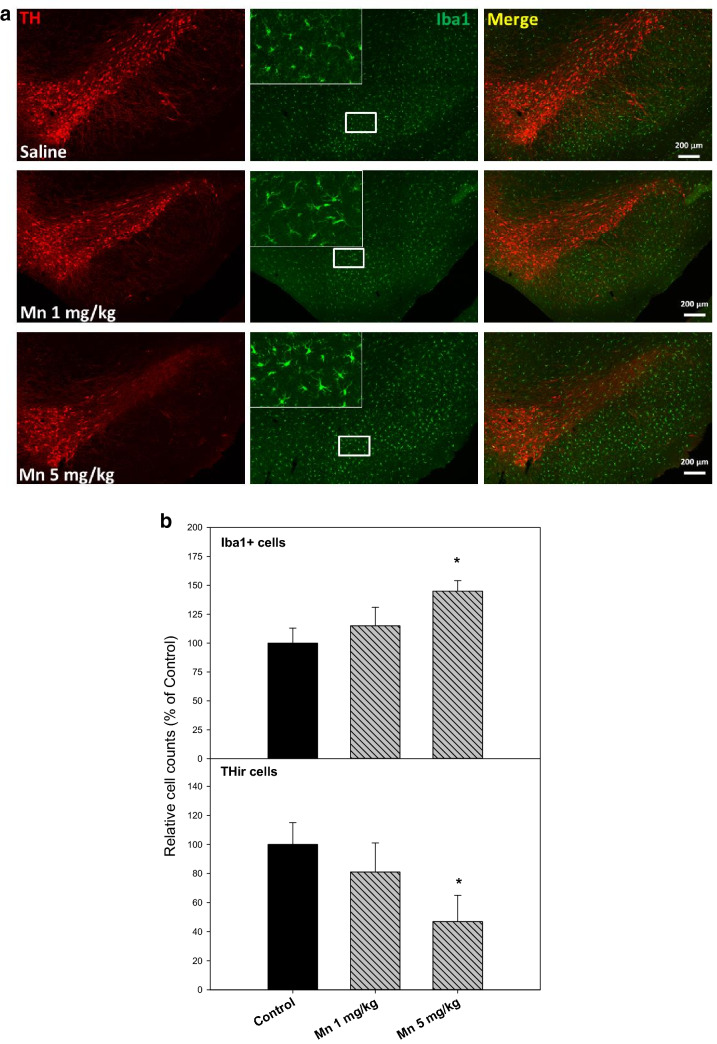
Chronic Mn-induced chronic neuroinflammation and loss of dopaminergic neurons. Adult male SD rats were given injections of saline, Mn^2+^ 1 and 5 mg/kg, i.p. every 10 days for 150 days. The brains were collected and SNpc region was double stained for dopaminergic neurons (THir-strain, red) and microglia (Iba1-stain, green). Representative photos were shown. **b** Relative cell counts in chronic Mn-induced chronic neuroinflammation and loss of dopaminergic neurons. Adult male SD rats were given injections of saline, Mn^2+^ 1 and 5 mg/kg, i.p. every 10 days for 150 days. The activation of microglia (Iba1+ cells) and loss of dopaminergic neurons (THir cells) were quantified. Data are mean + SEM of 3 rats. *Significantly different from Controls, p < 0.05

Subacute Mn^2+^ injections (30 days) produced chronic neuroinflammation and dopaminergic neuron loss in rats [25]. Whether chronic injection of Mn^2+^ every 10 days for 150 days could also produce similar pathology changes in rats was examined. As illustrated in Fig. 2a, 150 days after 15 injections of Mn^2+^ 1 mg and 5 mg/kg, Mn^2+^ induced microglia activation in a dose-dependent manner, characterized by hypertrophied morphology and intensified Iba1 staining (green). The enlarged photo showed ramified microglia and the numbers of microglia were increased by 15 and 45% after Mn^2+^ 1 mg and 5 mg/kg, respectively (Fig. 2b). Upon microglia activation, dopamine neurons loss was evidenced by reduced THir neurons (red). The numbers of THir cells were decreased by 19 and 53% after Mn^2+^ 1 mg and 5 mg/kg, respectively (Fig. 2b). These results demonstrated that Mn injections induced chronic neuroinflammation in rats causing delayed loss of THir neurons in SNpc, a brain region sensitive to Mn and LPS intoxication [25, 26]. It should be noted that in this study, 20% rats in the Mn^2+^ 5 mg/kg group died, which could have more severe injury than surviving rats.

### Chronic Mn Treatment Increased Inflammatory Gene Expression in the Brain

**Fig. 3:**
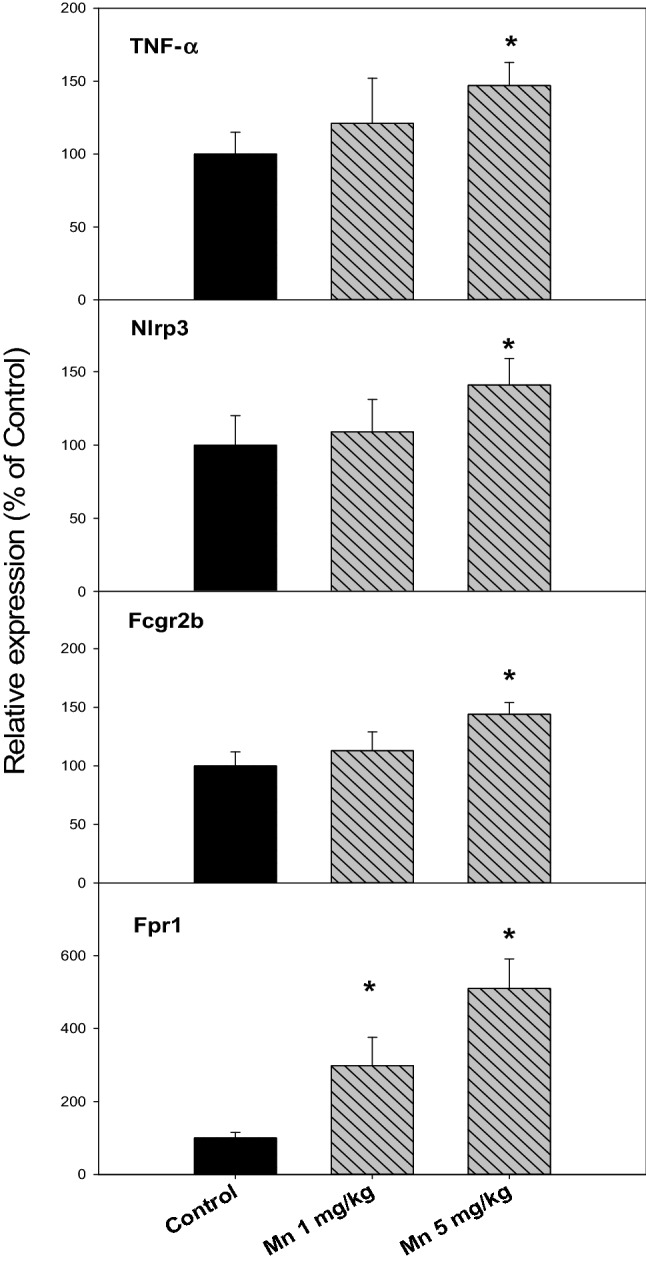
Inflammatory gene expressions in the SNpc-enriched midbrain. Rats were given injections of saline, Mn^2+^ 1 and 5 mg/kg, i.p. every 10 days for 150 days (15 injections), and the SNpc-enriched midbrain was isolated for RNA extraction, and the expression of inflammatory genes was examined via qPCR. Data are mean ± SEM of 5–7 rats. *Significantly different from Controls, p < 0.05

At the end of the experiment, brains were collected (n = 7, 7 and 5 for Control, Mn^2+^ 1 mg and 5 mg/kg, respectively). The SNpc-enriched midbrain was isolated to extract RNA for real-time RT-qPCR analysis (Fig. 3). The expression of TNFα in the SNpc-enriched midbrain was 100, 121 and 147% for Control, Mn^2+^ 1 mg/kg, and 5 mg/kg, respectively; The expression of inflammasome Nlrp3 in the SNpc-enriched midbrain was 100, 109 and 141% for Control, Mn^2+^ 1 mg/kg, and 5 mg/kg, respectively; The expression of Fcgr2b in the SNpc-enriched midbrain was 100, 113 and 144% for Control, Mn^2+^ 1 mg/kg, and 5 mg/kg, respectively; The expression of formyl peptide receptor 1 (Fpr1) was markedly increased (100, 298 and 510% for Control, Mn^2+^ 1 mg/kg, and 5 mg/kg, respectively). However, there were no changes in the expression of acute inflammatory mediators IL-1β and iNOS (data not shown).

### Chronic Mn Treatment Produced Hepatocyte Degeneration and Apoptotic Lesions

**Fig. 4:**
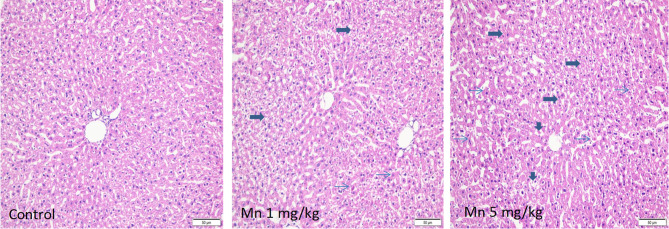
Representative H&E images of liver. Adult male SD rats were given injections of saline, Mn^2+^ 1 and 5 mg/kg, i.p. every 10 days for 150 days. Arrows indicate hepatocyte vacuolation and degeneration, and thin arrows indicate pink, condensed nuclei, indicative of apoptotic lesions. Magnitude × 100

Liver is a target of Mn toxicity [16, 25, 35]. Representative H&E microphotos are shown in Fig. 4. In livers of rats receiving Mn^2+^ 1 mg/kg, foci of liver degeneration and apoptotic lesions could be seen, but the liver injury was mild; In comparison, in livers of rats given high dose of Mn^2+^, widespread hepatocyte vacuolation and degeneration (arrows), apoptotic lesions (pink, condensed nuclei were observed (thin arrows), and focal necrosis (vertical arrowheads) could be observed.

### Chronic Mn Treatment Produced Inflammatory and Stress Gene Expression in the Liver

**Fig. 5:**
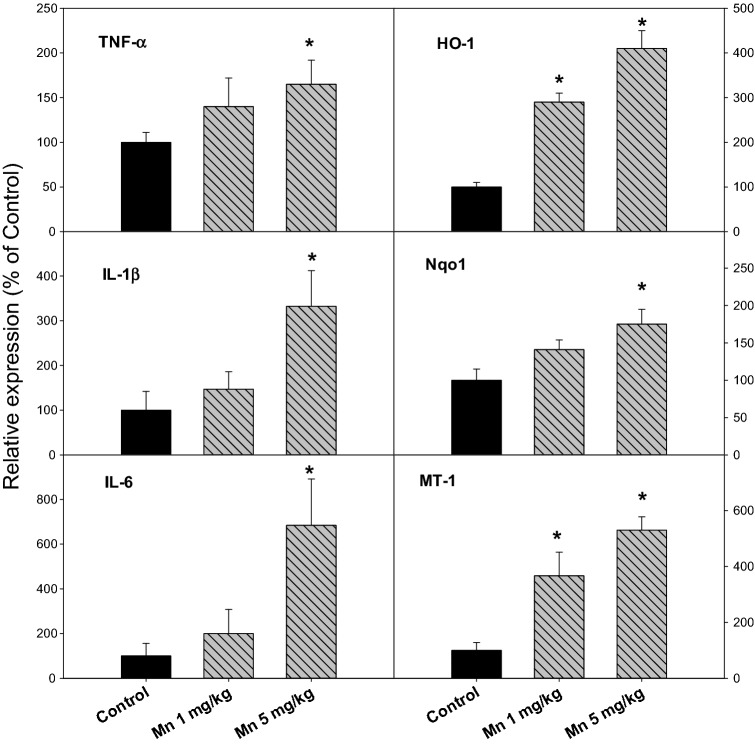
Pro-inflammatory gene and stress gene expression in the liver. Rats were given injections of saline, Mn^2+^ 1 and 5 mg/kg, i.p. every 10 days for 150 days (15 injections), and liver RNA was isolated, and subjected to qPCR analysis. Data are mean ± SEM of 8–10 rats. *Significantly different from Controls, p < 0.05

At the end of the experiment, livers were collected (n = 10, 10 and 8 for Control, Mn^2+^ 1 mg and 5 mg/kg, respectively). Total RNA was extracted for real-time RT-qPCR analysis (Fig. 5). For inflammatory cytokines, the expression of TNFα in the liver was 100, 116 and 155% for Control, Mn^2+^ 1 mg/kg, and 5 mg/kg, respectively; The expression of IL-1β was 100, 147 and 332% for Control, Mn^2+^ 1 mg/kg, and 5 mg/kg, respectively; the expression of IL-6 was 100, 200 and 684% for Control, Mn^2+^ 1 mg/kg, and 5 mg/kg, respectively. For stress genes, the expression of heme oxygenase-1(HO-1) was markedly increased (100, 290 and 410% for Control, Mn^2+^ 1 mg/kg, and 5 mg/kg, respectively), the expression of NAD(P)H:quinone oxidoreductase-1 (Nqo1) was 100, 141, and 175% for Control, Mn^2+^ 1 mg/kg, and 5 mg/kg, respectively; and the expression of metal-binding protein gene metallothionein-1 (MT-1) was 100, 367, and 530% for Control, Mn^2+^ 1 mg/kg, and 5 mg/kg, respectively.

### Chronic Mn Treatment Induced Hepatic Transporter Expressions

**Fig. 6:**
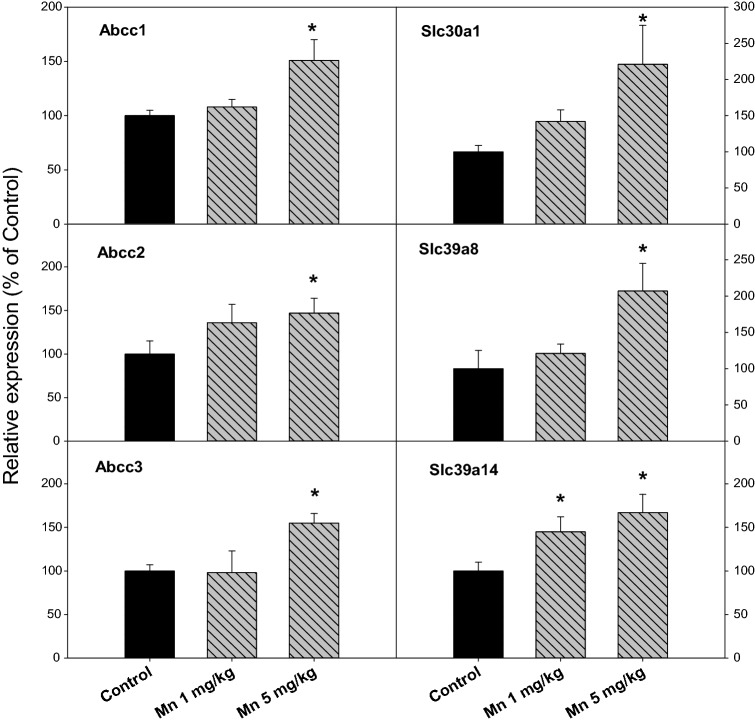
Expression of Abcc and Slc transporters. Rats were given injections of saline, Mn^2+^ 1 and 5 mg/kg, i.p. every 10 days for 150 days, and liver RNA was isolated, and subjected to qPCR analysis. Data are mean ± SEM of 8–10 rats. *Significantly different from Controls, p < 0.05

Mn is thought to be metabolized and removed by the liver [31, 35]. The expression of genes encoding hepatic transporters was further examined. Figure 6 illustrates the induction of hepatic transporters following chronic Mn exposure. Multidrug resistance proteins (MRPs) are main hepatic efflux transporters [20]. The expression of MRP1 (Abcc1) was 100, 108, and 151% for Control, Mn^2+^ 1 mg/kg, and 5 mg/kg, respectively; the expression of MRP2 (Abcc2) was 100, 136, and 147% for Control, Mn^2+^ 1 mg/kg, and 5 mg/kg, respectively; and the expression of MRP3 (Abcc3) was 100, 98, and 155% for Control, Mn^2+^ 1 mg/kg, and 5 mg/kg, respectively. The expression of ZnT1 (Slc30a1), Mn specific transporter Slc39a8 and Slc39a14 were also examined. The expression of ZnT1 (Slc30a1) was 100, 142, and 221% for Control, Mn^2+^ 1 mg/kg, and 5 mg/kg, respectively; the expression of Slc39a8 was 100, 121, and 207% for Control, Mn^2+^ 1 mg/kg, and 5 mg/kg, respectively; and the expression of Slc39a14 was 100, 145, and 167% for Control, Mn^2+^ 1 mg/kg, and 5 mg/kg, respectively.

## Discussion

The present study clearly demonstrated that chronic (150 days) Mn administration with longer intervals (10 days) between injections produced neurotoxicity in rats, as evidenced by impaired Rotarod activity, activation of microglia and loss of dopaminergic neurons in the SNpc region of the brain. These neurotoxicity effects were accompanied by increased expression of neuro-inflammation mediators. Chronic Mn also produced liver injury, with hepatocyte degeneration and apoptotic lesions. Molecular analysis revealed the enhanced expression of proinflammatary cytokines and stress protein genes. Furthermore, the expressions of hepatic transporters including Abcc families and Slc families were increased in an attempt to remove Mn from the liver. This study could add to our understanding of chronic Mn toxicity to the brain and liver.

In laboratory studies, most of experiments utilized consecutive injections of Mn. However, the half-life of Mn is relatively short [31], and the body has a strong recovery capability after cessation of Mn exposure (Cao et al., manuscript in preparation). In the present study we choose 10 days of recovery time between injections, in an attempt to give animals enough time to recover from Mn-induced acute stress, but the exposure lasted much longer (150 days) to mimic chronic progression of Manganism. The low dose of Mn^2+^ (1 mg/kg) caused mild abnormalities, while the high dose of Mn^2+^ 5 mg/kg caused 20% mortality after 140 days of administration, demonstrating a dose-dependent chronic Mn toxicity. The threshold of Mn toxicity is narrow, the increased dose renders it from an essential metal to a toxic metal [31]. Rats receiving the high dose of Mn had significantly decreased Rotarod activity, consistent with subacute Mn injections in our earlier observations [25]. Mn intoxication decreases spontaneous locomotor behavior (open field test) and muscle strength (weight test) [11]. Mn also induces non-motor behavior deficits such as the memory and recognition deficits in water maze and step-down tests [38]. The developmental (PND 8–12) exposure to MnCl_2_ (5–20 mg/kg) could result in behavioral deficits during adulthood (PND 60–65) [33]. Thus, behavioral impairments are important aspects of chronic Mn intoxication in humans [34, 35] and in experimental animals [11, 25, 33, 38].

Mn exposure specifically damage the basal ganglia in humans [1, 34, 35]. Activation of microglia and loss of THir neurons are the characters of Mn neurotoxicity either from intrastriatal injection [44] or from intraperitoneal injection [25]. The present immunofluorescent double staining of the SNpc clearly demonstrated that loss of THir neurons was accompanied by activation of microglia, indicating the key role of microglia activation in Manganism. Chronic microglial activation may be fueled either by Mn accumulation in the brain or by dying/damaged neurons characterized by enhanced TNFα expression [3]. Accumulating evidence has suggested that factors like damage-associated molecular patterns (DAMPs, including inflammasome Nlrp3) released by stressed and dying neurons are likely involved in self-propelling activation of microglia [6]. The increased expression Fcgr2b in neurons mediates cell-to-cell transmission of α-synuclein contributing to PD [7]. Formyl-peptide receptors (FPRs) are increasingly recognized for their expression in diverse host cell types to interact with chemotactic DAMPs contributing to inflammation and many pathophysiologic diseases [5]. Thus, overexpression of inflammatory mediates contributes to chronic microglia activation leading to neurodegeneration, loss of dopaminergic neurons, Parkinsonism, and Manganism.

Liver is a major target organ of Mn exposures [4, 14, 35, 40]. Chronic liver diseases further contribute to excessive accumulation of Mn in the brain leading to Mn hepatic encephalopathy [17, 23]. Liver dysfunction could lead to neurodegeneration with continued Mn exposure [40]. Compared to severe hepatotoxicity following acute and subacute exposed to Mn [16, 18], the liver injury observed in the present study was relatively mild, probably due to the longer time of recovery intervals (10 days) between injections. Nonetheless, at the high dose of Mn^2+^ 5 mg/kg, hepatocyte degeneration and apoptotic lesions and focal necrosis were evident. At molecular levels, the expression of pro-inflammatory cytokines, TNFα, IL-1β, and IL-6, were coordinately increased, indicating that chronic Mn induced inflammation to the liver [32]. Together with the inflammatory responses, acute-phase stress proteins were increased. HO-1 and Nqo1 are oxidative stress markers, but also indicators of the Nrf2 antioxidant pathway activation to combat with Mn-induced stress to rescue the liver [22]. MT-1 is a metal-binding protein, its upregulation plays important roles in metal detoxication, including Mn [21]. The induction of HO-1, Nqo1, and MT-1 in the liver can be envisioned as adaptive responses to Mn-induced oxidative stress and liver injury. It should be mentioned that chronic Mn exposure increased oxidative stress biomarkers in the brain and liver, raising the possibility of antioxidant interventions, which deserves further investigation.

Mn is mainly removed from the liver through bile [19], and multidrug resistant proteins (MRPs, ABCCs) play a critical role in the transport of xenobiotics from the liver to the bile [20]. In the present study, the increases of MRP transporters were observed in a Mn dose-dependent manner, which could be resulted from the activation of the Nrf2 pathway [22]. The increased MRPs transporters could help elimination of Mn into the bile as one of adaptive mechanisms. Slc30 subfamilies are implicated in zinc and Mn transport. For example, Slc30a10 (ZnT10) in the liver could regulate brain Mn concentration [42]. Slc39a8 (ZIP 8) and Slc39a14 (ZIP 14) are both involved in cellular transport of Mn, controlling tissue Mn levels [2, 41]. Upregulation of these MRPs and SLCs transporters could be envisioned as cellular adaptive mechanisms to remove Mn from the liver.

In summary, this study demonstrates that chronic Mn exposure with longer recover time between injections and longer exposure times produced activation of microglia, neuroinflammation and loss of dopaminergic neurons in the brain, but also produced inflammation in the liver leading to liver injury and activation of the Nrf2 antioxidant pathway and upregulation of efflux transporters as adaptive mechanisms against chronic Mn toxicity.

## Electronic supplementary material

Below is the link to the electronic supplementary material.

Supplementary file1 (DOCX 32 kb)

## Data Availability

All data are available upon request. N/A. N/A. All authors have read and approved the final version of the manuscript.
