# Supplementary material for: Chronic Manganese Administration with Longer Intervals Between Injections Produced Neurotoxicity and Hepatotoxicity in Rats
Source: Neurochem Res. 2020 Jun 2;45(8):1941–52. doi: 10.1007/s11064-020-03059-2 (PMC7378106; doi:10.1007/s11064-020-03059-2)
Supplement: Supplementary file 1 — Supplementary file1 (DOCX 32 kb) [file 11064_2020_3059_MOESM1_ESM.docx]

Supplementary Table 1： Primer sequences for qPCR
